# Integrating spatial transcriptomics and bulk RNA-seq: predicting gene expression with enhanced resolution through graph attention networks

**DOI:** 10.1093/bib/bbae316

**Published:** 2024-07-03

**Authors:** Sudipto Baul, Khandakar Tanvir Ahmed, Qibing Jiang, Guangyu Wang, Qian Li, Jeongsik Yong, Wei Zhang

**Affiliations:** Department of Computer Science, University of Central Florida, Orlando, FL 32816, United States; Department of Computer Science, University of Central Florida, Orlando, FL 32816, United States; Department of Computer Science, University of Central Florida, Orlando, FL 32816, United States; Houston Methodist Research Institute, Weill Cornell Medical College, Houston, TX 77030, United States; Department of Biostatistics, St. Jude Children’s Research Hospital, Memphis, TN 38105, United States; Department of Biochemistry, Molecular Biology and Biophysics, University of Minnesota Twin Cities, Minneapolis, MN 55455, United States; Department of Computer Science, University of Central Florida, Orlando, FL 32816, United States

**Keywords:** spatial transcriptomics, whole slide image, spot-level gene expression estimation, Graph Attention Network

## Abstract

Spatial transcriptomics data play a crucial role in cancer research, providing a nuanced understanding of the spatial organization of gene expression within tumor tissues. Unraveling the spatial dynamics of gene expression can unveil key insights into tumor heterogeneity and aid in identifying potential therapeutic targets. However, in many large-scale cancer studies, spatial transcriptomics data are limited, with bulk RNA-seq and corresponding Whole Slide Image (WSI) data being more common (e.g. TCGA project). To address this gap, there is a critical need to develop methodologies that can estimate gene expression at near-cell (spot) level resolution from existing WSI and bulk RNA-seq data. This approach is essential for reanalyzing expansive cohort studies and uncovering novel biomarkers that have been overlooked in the initial assessments. In this study, we present STGAT (Spatial Transcriptomics Graph Attention Network), a novel approach leveraging Graph Attention Networks (GAT) to discern spatial dependencies among spots. Trained on spatial transcriptomics data, STGAT is designed to estimate gene expression profiles at spot-level resolution and predict whether each spot represents tumor or non-tumor tissue, especially in patient samples where only WSI and bulk RNA-seq data are available. Comprehensive tests on two breast cancer spatial transcriptomics datasets demonstrated that STGAT outperformed existing methods in accurately predicting gene expression. Further analyses using the TCGA breast cancer dataset revealed that gene expression estimated from tumor-only spots (predicted by STGAT) provides more accurate molecular signatures for breast cancer sub-type and tumor stage prediction, and also leading to improved patient survival and disease-free analysis. Availability: Code is available at https://github.com/compbiolabucf/STGAT.

## Introduction

Advancements in genome sequencing technologies have made RNA-seq gene expression data affordable and accessible for biomedical research [1, 2]. Correspondingly, Hematoxylin and Eosin (H&E) stained Whole Slide Images (WSI) are now easily obtainable [3]. Integrating RNA-seq gene expression and WSI data from the same cohort can enhance disease diagnosis accuracy. However, bulk RNA-seq data lacks detailed tissue environment information found in WSI cross-sections and may include expressions of non-disease cells that could affect prediction outcomes [4]. In this regard, the information from single-cell RNA-seq (scRNA-seq) proves to be valuable. For example, the type, density, and location of immune cells within tumor samples are crucial for predicting cancer sub-types as they reveal latent disease information [5]. Therefore, examining gene expression at or near the cell level, along with location information, aids in diagnosing complex biological systems and structures, particularly in treating compound diseases like cancer [6, 7]. Spatial transcriptomics data, comprising spot-level information, has opened the path for meticulous analysis of tissue sections and offers a potential connection between affordable bulk RNA-seq and cell-level gene expression data, enabling the retention of location and cell-type information [8, 9]. By using spatial transcriptomics, a WSI of a tissue cross-section can be divided into multiple gene expression fragments known as spots. Each spot represents a few cells and contains gene expression profiles with positional information [10, 11].

Spatial transcriptomics has proven successful in analyzing various cancer tissues, including breast [11], prostate [12], melanoma [13], pancreas [14] and carcinoma [15]. It has also been applied to study diseased tissues such as Alzheimer’s disease [16] and gingivitis [17], as well as healthy tissues like the mouse olfactory bulb [11], human heart [18], spinal cord [19] and brain [20]. Despite these advancements, the applicability of spatial transcriptomics data to large cohort studies is often limited because it is relatively expensive, labor-intensive and time-consuming [6, 9, 21]. Additionally, the technology is relatively new compared to bulk RNA-seq. Hence, spatial transcriptomics data is unavailable in most situations. Nevertheless, there are several large cohort disease studies (e.g. TCGA [22]) that provide both bulk RNA-seq expression for a sample and the corresponding WSI [1, 2]. An estimation of spot-level gene expression from bulk RNA-seq and WSI in these large cohort studies can be leveraged to uncover more precise molecular mechanisms at a near-cellular level for different diseases. This study focuses on developing such an approach to estimate spot-level gene expression from bulk RNA-seq expression.

Several computational methods have been developed to analyze spatial transcriptomics data and unveil latent characteristics of human tissues and organs. These methods facilitate the analysis of spatial expression patterns [23, 24], determination of cell-type composition [25, 26], investigation of cell-to-cell communication [27, 28], clustering into spatial domains [29, 30] and generation of spatial embedding representations [31]. Furthermore, attempts have been made to predict gene expression at the spot-level using methods such as ST-Net [32] and HistoGene [33]. A recent method named iSTAR [34] aims to predict gene expression profiles at the pixel level using HViT (Hierarchical Vision Transformer). However, these approaches do not fully exploit the rich information contained in the data. ST-Net and iSTAR process spot images (patches) independently without considering location information, while HistoGene incorporates spatial coordinates but disregards internal 2D visual features of the images. Graph Neural Networks [35, 36] show promise in addressing these limitations and may offer valuable insights in this context.

In recent years, network-based computational methods, capable of extracting topological information from large-scale biological data, have shown great promise in solving complex problems, including precision oncology [37], drug sensitivity analysis [38] and single-cell RNA-seq analysis [39]. The spatial information present in spatial transcriptomics data can be harnessed to construct a network. The coordinates of a spot provide insight into its location within the tissue section and offer contextual information about the surrounding environment, leading to improved disease diagnosis [29, 40, 41]. For instance, spots infected with a specific sub-type of cancer may exhibit spatial proximity and influence each other’s behavior. Consequently, a network can be generated using spot location information, with closer spots being connected to one another. Hist2ST [42] and THItoGene [43], which employ the graph learning algorithms, have demonstrated the effectiveness of spatial networks derived from spatial transcriptomics data. They outperform previous models (i.e. ST-Net and HistoGene) in gene expression prediction tasks. However, both of them predict only a limited number of gene expression profiles, which restricts the downstream tasks utilizing such expression data. Additionally, their applicability is limited to spatial transcriptomics data only. The performance of Hist2ST and THItoGene suggests that the utilization of network information available in spatial transcriptomics can significantly impact the results. Graph Attention Network (GAT) [44] can efficiently extract topological information from a network and has shown success in various biological tasks, including cancer sub-type prediction [45], drug-target interaction prediction [46], tissue structure prediction from spatial data [40], drug-microbe interaction prediction [47], gene essentiality prediction [48] and single-cell RNA-seq based dimensionality reduction [49]. We aim to extend this exemplary performance of GAT to spatial transcriptomics. GAT leverages the attention mechanism [50] to accumulate information from neighboring nodes in a network, with certain neighbors receiving more attention than others. This mechanism enables the network to focus on relevant and informative connections within the data.

Motivated by the need to obtain spot-level gene expression and drawing inspiration from the success of the GAT model, we present STGAT, a novel machine learning framework designed to predict gene expression profiles at the spot-level. STGAT utilizes spot images extracted from WSIs and estimates the gene expression for each spot. The framework makes use of the limited availability of spatial transcriptomics data to train STGAT, mapping the spatial information of each spot to gene expression. The trained model is then transferred to bulk RNA-seq data to estimate spot-level gene expression. This framework enables detailed downstream analysis tasks by allowing researchers to focus on specific regions of interest within a tissue sample. To evaluate the performance of STGAT, we conducted tests on a large-scale cancer study (i.e. TCGA [22]), which encompasses tens of thousands of cancer samples, providing both WSI and bulk RNA-seq gene expression data. Our hypothesis suggests that gene expression originating from tumor-only spots within a WSI exhibits a stronger correlation with the disease phenotype, thereby offering more accurate molecular signals for predicting cancer sub-types compared to bulk RNA-seq data. Consequently, our objective is to classify spots from a WSI into tumor and non-tumor categories and estimate the gene expression of each tumor spot. Subsequently, gene expression exclusively derived from tumor-only spots is utilized for downstream analyses, including biomarker identification, cancer sub-type prediction and patient survival prediction.

## Materials and methods

**Figure 1:**
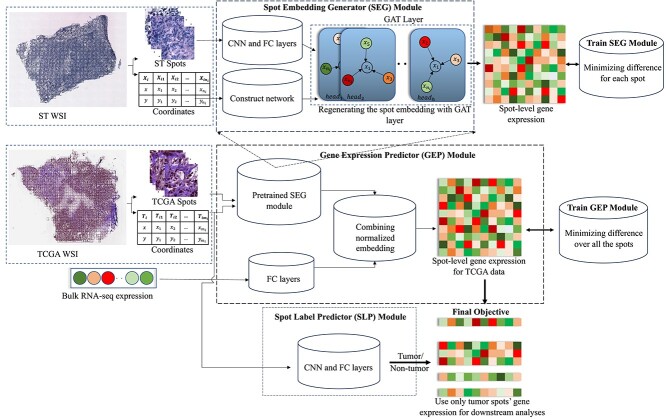
An overall illustration of the proposed framework. The SEG produces embeddings for spot images extracted from a WSI. The GEP then estimates spot-level gene expression profiles for the WSI by leveraging the embeddings generated by SEG and the bulk RNA-seq gene expression data of the corresponding WSI. The SLP classifies each spot on the WSI as either tumor or non-tumor. In this study, SEG is trained and evaluated on spatial transcriptomics data, GEP is trained and evaluated on TCGA data, and SLP is trained on spatial transcriptomics data and applied to TCGA data.

We introduce the STGAT (Spatial Transcriptomics Graph Attention Network) model in this section. Firstly, we provide an overview of the model architecture, outlining its key components (Fig. 1), followed by a detailed description of each component. Next, we describe the datasets utilized for training and evaluating STGAT, along with the pre-processing steps applied to these datasets. Lastly, we elucidate the training procedure employed for the various components of the model.

### Model framework

The primary objective of the STGAT model is to generate spot-level gene expression for WSIs using available bulk RNA-seq gene expression data. The underlying assumption is that identifying tumor spots and using only these spots can improve downstream task performance by reducing noise introduced by other spots that are irrelevant to the downstream analysis. The STGAT model consists of three fundamental modules: (i) Spot Embedding Generator (SEG), (ii) Gene Expression Predictor (GEP) and (iii) Spot Label Predictor (SLP). Initially, SEG is trained on spatial transcriptomics data to map spots from spatial transcriptomics images to their corresponding gene expression values. Once trained, the SEG is transferred to bulk RNA-seq data to generate spot-level gene expression through the GEP module. The GEP module consists of two components: the pretrained SEG and fully connected (FC) layers. Spots generated from WSIs associated with bulk RNA-seq data are fed into the pretrained SEG to obtain spot embeddings. These spot embeddings, combined with bulk RNA-seq gene expression data processed through the FC layers, are used to estimate spot-level gene expression profiles for the WSI. Finally, the SLP module, trained on spatial transcriptomics data, is used to classify spots as either tumor or non-tumor. The labels produced by the SLP module for a WSI are then used to select only the gene expression profiles of tumor spots from the spot-level gene expression generated by the GEP module. Tables S1, S2 and S3 in the Supplementary document include the mathematical notations utilized in the Methods section, the number of different blocks used in each module and the composition of each block, respectively.

#### Spot Embedding Generator

The SEG module, depicted at the top of Fig. 1, generates embeddings for spots with dimensions of $224\times 224$. Trained on spatial transcriptomics data, the SEG module starts with six Convolutional Neural Network (CNN) blocks followed by an FC layer. These CNN blocks are essential for extracting features from the spot images, with deeper layers capturing specific details of the spots. To avoid losing the 2D contextual information of the spots, we have limited the model to six layers. The intermediate FC layer prepares the embeddings generated by the final CNN block for input into the GAT layer.

Let’s consider there are $v$ spatial transcriptomics images, denoted as $\mathbf{X} = [\mathbf{X}_{1}, \mathbf{X}_{2}, \ldots , \mathbf{X}_{v}]$. Additionally, each $\mathbf{X}_{i}$ image contains $g_{i}$ spots, and let $p$ represent the number of genes to be estimated within each spot in each image. These spot images are fed into the first CNN block in mini-batches. Each CNN block comprises a convolutional layer, a Rectified Linear Unit (ReLU) activation function and a MaxPooling layer, except for the first CNN block.


(1) \begin{align*} & \mathbf{E}^{(1)}_{ij} = MaxPool(ReLU(Convolution(\mathbf{X}_{ij}))) \end{align*}

(2) \begin{align*} & \mathbf{E}^{(2)}_{i} = ReLU(\mathbf{WE}^{(1)}_{i}+\mathbf{B}) \end{align*}

Following Equation (1), the output from the final CNN block for the $j$th spot is represented by $\mathbf{E}^{(1)}_{ij}\in \mathbb{R}^{e_{c}}$, where $e_{c}$ is the length of the embedding vector. $\mathbf{E}^{(1)}_{i}\in \mathbb{R}^{g_{i}\times e_{c}}$ denotes the embeddings for all $g_{i}$ spots in $\mathbf{X}_{i}$, which is passed through the intermediate FC layer according to Equation (2) to generate $\mathbf{E}^{(2)}_{i}\in \mathbb{R}^{g_{i}\times e_{c}}$.

Subsequently, $\mathbf{E}^{(2)}_{i}$ undergoes processing via a GAT layer. This layer makes use of the adjacency matrix, denoted as $\mathbf{A}_{i}$ (excluding self-connections), which is derived from the distance matrix of the image $\mathbf{X}_{i}$ (as detailed in Section *Adjacency matrix*), to establish the underlying graph structure. Our hypothesis suggests that when the majority of a spot’s neighboring points are classified as tumor spots, there is a high possibility that the spot itself is also a tumor spot. We employ two distinct attention coefficients: neighbor attention, denoted as $\alpha _{n}$, and self-attention, denoted as $\alpha _{s}$, with the aim of directing the model to place less emphasis on individual spots and prioritize information from their neighbors. This approach facilitates the channeling of the characteristics of neighboring spots into the spot’s embedding. For the $j$th spot, the neighbor attention coefficient from the $k$th neighbor can be calculated as follows: 


(3) \begin{align*}& \alpha_{n_{ijk}} = ReLU\left(\mathbf{a}_{s}\mathbf{W}_{s}\mathbf{E}^{(2)}_{ij} + \mathbf{a}_{n}\mathbf{W}_{n}\mathbf{E}^{(2)}_{ik}\right)\end{align*}

where $\mathbf{a}_{s}, \mathbf{a}_{n}\in \mathbb{R}^{e_{a}}$ are the self and neighbor attention vectors, respectively, with $e_{a}$ representing the embedding length of the GAT. $\mathbf{W}_{s},\mathbf{W}_{n}\in \mathbb{R}^{e_{a}\times e_{c}}$ are weight matrices of self and neighbor attention. The neighbor attention coefficient vector $\mathbf{\alpha }_{n_{ij}}\in \mathbb{R}^{g_{i}}$ for the $j$th spot is updated to retain values only for connections present in $\mathbf{A}_{i}$, while others are set to zeros. $\mathbf{\alpha }_{n_{ij}}$ is then passed through the Softmax function for normalization to ensure model compatibility with different graph structures generated from various images. Next, the self-attention coefficient is computed from the neighbor attention coefficient vector using the formula $\mathbf{\alpha }_{s_{ij}}=\frac{1}{1+\sum _{k\in \mathcal{N}_{ij}}\alpha _{n_{ijk}}}$, where $\mathcal{N}_{ij}$ represents the set of the neighbors for the $j$th spot.

Finally, the spot embedding for the $j$th spot is determined by Equations (4) and (5): 


(4) \begin{align*} & \mathbf{E}^{^{\prime}(3)}_{ij} = f\left(\mathbf{\alpha}_{s_{ij}}\mathbf{W}^{^{\prime}}_{s}\mathbf{E}^{(2)}_{ij} + \sum_{k\in\mathcal{N}_{ij}}\mathbf{\alpha}_{n_{ijk}}\mathbf{W}^{^{\prime}}_{n}\mathbf{E}^{(2)}_{ik}\right) \end{align*}

(5) \begin{align*} & \mathbf{E}^{(3)}_{ij} = \Arrowvert_{l=1}^{h} f\left(\mathbf{\alpha}_{s_{ijl}}\mathbf{W}_{s_{l}}\mathbf{E}^{(2)}_{ij} + \sum_{k\in\mathcal{N}_{ij}}\mathbf{\alpha}_{n_{ijkl}}\mathbf{W}_{n_{l}}\mathbf{E}^{(2)}_{ik}\right) \end{align*}

where $\mathbf{E}^{^{\prime}(3)}_{ij}\in \mathbb{R}^{e_{a}}$ represents the spot embedding from a single head, $\mathbf{E}^{(3)}_{ij}\in \mathbb{R}^{e}$ is the spot embedding obtained from $h$ heads ($e=he_{a}$) and $f$ denotes a non-linear function.

Spot embedding $\mathbf{E}^{(3)}_{i}$ is then passed through two FC layers to produce spot-level gene expressions, $\mathbf{Y}^{(1)}_{i}\in \mathbb{R}^{g_{i}\times p}$, for each spatial transcriptomics image $\mathbf{X}_{i}$. The purpose of the FC layers is to project the embeddings of the spots onto gene expression profiles, which are necessary for training the SEG module. The final FC layers are omitted from the pretrained SEG when it is used to generate spot embeddings for WSIs as part of the GEP module.

#### Gene Expression Predictor

The GEP module, depicted in the middle panel of Fig. 1, is designed to predict spot-level gene expression for WSI samples. Let $\mathbf{T} = [\mathbf{T}_{1}, \mathbf{T}_{2}, \ldots , \mathbf{T}_{t}]$ represents the set of WSIs, where $\mathbf{T}_{i}$ denotes the $i$th WSI containing $m_{i}$ spots. Furthermore, $\widetilde{\mathbf{U}}_{i} \in \mathbb{R}^{p}$ represents the corresponding bulk gene expression in the set $\widetilde{\mathbf{U}} = [\widetilde{\mathbf{U}}_{1}, \widetilde{\mathbf{U}}_{2}, \ldots , \widetilde{\mathbf{U}}_{t}]$, with $p$ denoting the number of genes in the bulk RNA-seq gene expression data. Spots are initially generated from $\mathbf{T}_{i}$ and input into the pretrained SEG to produce an embedding, $\mathbf{E}^{(3)}_{i}\in \mathbb{R}^{m_{i}\times e}$. Concurrently, $\widetilde{\mathbf{U}}_{i}$ undergoes processing through the first component of GEP, which consists of two FC layers, resulting in the generation of a WSI embedding, $\mathbf{Z}^{(1)}_{i}\in \mathbb{R}^{e}$. To align $\mathbf{E}^{(3)}_{i}$ and $\mathbf{Z}^{(1)}_{i}$ in a compatible feature space, two learnable weight parameters, $\mathbf{W}_{spots}$ and $\mathbf{W}_{bulk}\in \mathbb{R}^{e\times e}$, are multiplied with them, as shown in Equation (6):


(6) \begin{align*}& \mathbf{E}^{(4)}_{i} = \mathbf{W}_{spots}\mathbf{E}^{(3)T}_{i}, \mathbf{Z}^{(2)}_{i} = \mathbf{W}_{bulk}\mathbf{Z}^{(1)T}_{i}.\end{align*}

Then Z-score normalization is applied to $\mathbf{E}^{(4)}_{i}$ and $\mathbf{Z}^{(2)}_{i}$. $\mathbf{Z}^{(2)}_{i}$ is broadcast and added to $\mathbf{E}^{(4)}_{i}$ to generate $\mathbf{Z}^{(3)}_{i}\in \mathbb{R}^{e \times m_{i}}$ in Equation (7).


(7) \begin{align*}& \mathbf{Z}^{(3)}_{i}= \mathbf{E}^{(5)}_{i} + Broadcast\left(\mathbf{Z}^{(2)T}_{i}\right)\end{align*}

Lastly, $\mathbf{Z}^{(3)}_{i}$ is passed through three FC layers to generate the spot-level gene expression for a single WSI, given by $\mathbf{Y}^{(2)}_{i}\in \mathbb{R}^{m_{i}\times p}$. During training, the average expression of all spots within a particular image is considered as its bulk gene expression, which is then compared to the actual bulk RNA-seq gene expression. The SEG module is pretrained on spatial transcriptomics data. The pretrained SEG and GEP are utilized to generate spot-level gene expression profiles for WSIs. In the final stage of our framework, the SLP is employed to classify spots as either tumor or non-tumor, enabling the use of only tumor spots’ gene expression data for downstream tasks.

#### Spot Label Predictor

As mentioned above, SLP predicts whether a spot is tumor or non-tumor by feeding spot images to it. SLP contains five CNN blocks (similar to Equation (1)), followed by a Maxpool2D layer, two FC layers (similar to Equation (2)) and a Softmax activation function at the end. SLP is trained on spatial transcriptomics data, which contains tumor/non-tumor label information for each spot. The pretrained SLP is then used to predict the label of the WSI spots, and only the tumor spots are used to compute the average gene expression of a WSI image for downstream tasks.

### Training procedure

The STGAT framework comprises three separate training processes for its three modules implemented using *Pytorch* [51]. Firstly, the SEG module is trained using spatial images from the spatial transcriptomics data. The spots generated from these images are input into the SEG module, which, in turn, produces gene expression predictions for all the spots. The training objective for the SEG module is based on the Mean Squared Error (MSE) loss, calculated as the MSE between the predicted spot-level gene expression, denoted as $\mathbf{Y}^{(1)}_{i}$ for the $i$th spatial image, and the corresponding true gene expression, denoted as $\widetilde{\mathbf{Y}}^{(1)}_{i}$ as defined in Equation (8). This MSE loss serves as the objective function for training the SEG module.


(8) \begin{align*}& \mathcal{L}_{MSE}=\left\Vert\widetilde{\mathbf{Y}}^{(1)}_{i}-\mathbf{Y}^{(1)}_{i}\right\Vert_{2}^{2}\end{align*}

After completing the training process, the last two FC layers of the SEG module are removed to extract the spots’ embeddings directly from the GAT layer.

Next, the GEP module is trained using TCGA WSIs (i.e. images). For each TCGA image, the spots generated from the $i$th image are fed into the pretrained SEG to obtain the corresponding spots’ embeddings. Simultaneously, the bulk RNA-seq gene expression data of the TCGA image is processed through two FC layers. The two representations obtained from the spots and bulk RNA-seq gene expression are integrated following Equations (6) and (7), as discussed in Section *Gene Expression Predictor*. Subsequently, spot-level gene expression, denoted as $\mathbf{Y}^{(2)}_{i}$, is generated for the TCGA image, and the average gene expression of all spots, represented as $\mathbf{U}_{i}\in \mathbb{R}^{p}$, is computed. The MSE loss between $\widetilde{\mathbf{U}}_{i}$ (the true bulk RNA-seq gene expression) and $\mathbf{U}_{i}$ is calculated in a manner similar to Equation (8), serving as the objective function for training the GEP module.

Finally, the SLP module is trained using the spatial image spots to determine the probability of each spot being tumor or non-tumor tissue. Given the imbalanced class labels in the spatial transcriptomics data, Youden’s Index [52] is employed to identify a threshold value for classifying each spot. Subsequently, the SLP module is trained with the objective of maximizing the $F_{1}$ score, which measures the harmonic mean of precision and recall, by comparing the true class labels with the predicted class labels for each spatial image.

### Data processing

#### Datasets

Spatial transcriptomics data, along with TCGA data, serve as critical components for training and assessing the proposed STGAT framework. The evaluation process involves the utilization of two distinct breast cancer spatial transcriptomics datasets. The first dataset is the human breast cancer in situ transcriptomics dataset, hereafter referred to as the ‘breast cancer dataset,’ was sourced from the Mendeley Data website [53]. The second dataset is the HER2-positive breast cancer dataset, hereafter referred to as the ‘HER2+ dataset,’ was obtained from the Zenodo data repository [54]. Additionally, TCGA Breast Invasive Carcinoma (BRCA) data [55] is employed to train the GEP module and to facilitate subsequent tasks related to gene expression prediction.

The ‘breast cancer dataset’ comprises a total of 68 spatial images, with each image containing around 1000 spots. For every spot within each image, there are 13 776 gene expression profiles available. It is important to note that spatial location and label information (tumor/non-tumor) is only accessible for approximately 250 to 700 spots within each image, while the remaining spots are considered as background and are not utilized in experimental procedures. In this study, 42 samples from the ‘breast cancer dataset’ are designated for training, while the remaining 26 samples are allocated for testing purposes. The ‘HER2+ dataset’ is constituted of 36 samples and the number of spots per sample within this dataset ranges from 177 to 712. Importantly, all samples in the ‘HER2+ dataset’ are exclusively used for testing. The tumor and non-tumor spots labeled by pathologists are utilized as the ground truth for the purpose of training the model.

The WSIs for the TCGA BRCA data were obtained from the Genomic Data Commons Data Portal [56]. The associated bulk RNA-seq data pertaining to the same patient samples were retrieved from the UCSC Xena Hub [57]. Clinical information for these patient samples was collected from cBioPortal [58]. The model was trained on 60 BRCA patient samples and tested on 349 BRCA patient samples with available clinical information.

#### Splitting image patches from WSI

Usually, the size of WSIs is very large. Additionally, using WSI images increases the computational complexity of the model by many folds [59]. Hence, the WSI is split, or specific regions of interest are selected from the image for processing to serve a specific purpose in a study [60–62]. In the spatial transcriptomics data, spatial spots are extracted from WSI using location information provided in the data. A region of $112$ pixels on both sides of the $(x,y)$ coordinates (considered as the center of the spot) is cropped from the WSI to obtain the spot. However, for TCGA WSIs, lacking location information, an initial division of the WSI into spots of dimensions $512\times 512$ pixels is performed. Subsequently, for each spot, the count of pixels with a mean RGB (Red, Green, Blue channels) value less than $220$ is determined. If this count is less than half of the spot’s pixels (i.e. $512\times 512/2$), the spot is classified as background; otherwise, it is considered a valid spot. Valid spots are then reshaped to $224\times 224$ pixels, and the corresponding row and column values are used as the spot’s $(x,y)$ coordinates for constructing the adjacency matrix.

#### Adjacency matrix

In the STGAT framework, an important component is the GAT layer, which is designed to leverage the spatial location information inherent in spatial transcriptomics data. This spatial context is conveyed to the GAT layer through an adjacency matrix of the spots network. For each sample image in spatial transcriptomics data or TCGA data, a spots network is constructed based on the coordinates ($x$, $y$) of the spots in each image. The Euclidean distance between pairs of spots is computed, as outlined by the following equation:


\begin{align*} &\sqrt{(x_{i}-x_{j})^{2}+(y_{i}-y_{j})^{2}}\end{align*}

where $i,j$ denote distinct spots. For a given spot, all computed distance values to other spots are normalized by dividing each value by the maximum among all such distances. Subsequently, only the connections where the distance values fall below a specified threshold are set to 1, signifying that the connection is retained, while all other connections are set to 0. This process is repeated for all spots, resulting in the construction of the binary adjacency matrix. Finally, any self-connection is eliminated by setting the diagonal entries of the matrix to 0.

### Baseline methods

To assess the performance of STGAT in predicting spot-level gene expression for spatial transcriptomics data, three state-of-the-art baseline methods were employed: (1) Hist2ST [42], (2) HisToGene [33] and (3) THItoGene [43]. However, all of them lack the capacity to directly predict spot-level gene expression from bulk RNA-seq gene expression data. Therefore, the evaluation of gene expression produced by STGAT on TCGA data is conducted through a comparison with true gene expression values.

A Vision Transformer (ViT) [63] is employed by HisToGene for the gene expression estimation task in the context of spatial transcriptomics data. The process begins by segmenting the spot image patches from WSIs based on their spatial coordinates. The embedding of spot image patches, along with positional embeddings, is aggregated using a modified ViT. Subsequently, multi-head attention layers are utilized to generate hidden embeddings, which are then fed into a Multilayer Perceptron for the prediction of gene expression.

Hist2ST employs three modules, namely, Convmixer, Transformer and GNN, to predict the gene expression for each sequenced spot. The Convmixer module is responsible for capturing the 2D relationships within the spot image patch, while the Transformer module extracts global spatial dependencies. The GNN module leverages the relationships between the spots through the utilization of the GraphSAGE algorithm [64]. The learned features integrated from these modules are subsequently utilized to predict gene expression, employing the zero-inflated negative binomial distribution.

THItoGene leverages four modules with specific functions for the gene expression prediction task. The Dynamic Convolution [65] module extracts and enhances features from the histological image, the Efficient-CapsNet [66] module captures spatial relationships and hierarchical structures among features, the ViT [63] module attempts to extrapolate long-range dependencies in the images, and the Graph Attention Network [44] module learns interactions between spatially neighboring spots.

## Experiments

To evaluate the performance of STGAT and the reliability of the estimated gene expression, we conducted comprehensive experiments in two main domains. The first set of experiments focused solely on spatial transcriptomics data, thereby assessing the efficacy of the SEG component of the model. The second set of experiments involved WSIs and the corresponding bulk RNA-seq data in TCGA, demonstrating STGAT’s ability to transfer acquired knowledge from the spatial domain to existing large-scale cancer studies. The impact of gene expression estimation by STGAT is substantiated through downstream analysis tasks. Section S1 in the Supplementary document explains the evaluation metrics used for the experiments.

### Evaluation with spatial transcriptomics data only

In the STGAT framework, the SEG module plays a pivotal role as it generates gene expression at the level of individual spots. To assess the reliability of the estimated gene expression, we calculate the correlation between the true gene expression and the predicted gene expression for each spot within every spatial image. This helps us assess how well our predictions align with the real gene expression patterns.

#### Estimated gene expression correlated with the ground truth

**Figure 2:**
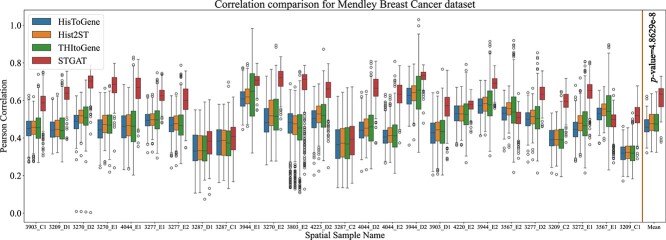
Experimentation on the ‘breast cancer dataset.’ The boxplots represent the correlation between the true gene expression and the predicted gene expression by STGAT and the baseline models for the 26 test samples of the ‘breast cancer dataset.’

The SEG component within the STGAT framework and the two baseline methods were trained on the 42 samples of the ‘breast cancer dataset’ and predicted the gene expression at the spot-level for the remaining 26 samples. The correlations and Mean Squared Error (MSE) between the true expression and estimated gene expression are presented in Fig. 2 and Supplementary Fig. S1, along with the average correlation score and average MSE loss across all samples, respectively. It is evident that STGAT significantly outperforms all the baseline methods. Notably, Hist2ST and THItoGene, which exploit a network structure constructed using spot location information, exhibits superior performance compared to HistToGene. This observation highlights the advantageous nature of integrating spatial information for gene expression prediction. Furthermore, the synergy between the attention mechanism and spatial information within the SEG module allows for the enhanced extraction of nuances of spatial transcriptomic data, contributing to the superior performance of STGAT. In a GAT layer, the attention mechanism and spatial information work cohesively to enhance results. The spatial information enables the model to identify neighbors for a target node, while the attention mechanism assigns significance to these neighbors by allocating attention coefficients. This process ensures a focus on the most crucial neighbors while disregarding the rest.

**Figure 3:**
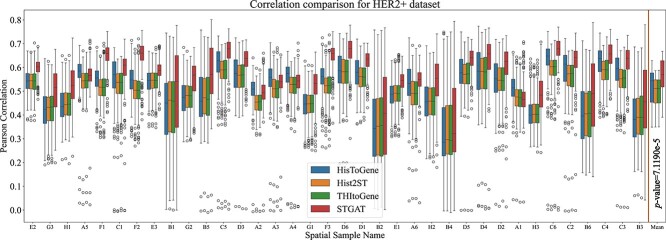
Experimentation on the ‘HER2+ dataset.’ The figure illustrates the correlation between the true gene expression and the predicted gene expression of the samples in the ‘HER2+ dataset’ by training STGAT and baseline models on the ‘breast cancer dataset.’

Motivated by these compelling results, we expanded our investigation to the ‘HER2+ dataset.’ We directly applied the STGAT model and the baseline models, previously trained on the ‘breast cancer dataset,’ to the 36 samples of this new dataset. The outcomes are illustrated in Fig. 3 and Supplementary Fig. S2. It is evident that STGAT consistently outperforms the baselines in terms of correlation and MSE loss across the majority of the samples. The strong correlation and lower MSE loss between gene expression predicted by STGAT and the real gene expressions gives us confidence in using the predicted gene expression at the spot-level for downstream analysis tasks.

To evaluate STGAT’s performance from a different perspective, we experimented with Kyoto Encyclopedia of Genes and Genomes [67] pathways. Supplementary Fig. S3 displays the top twenty pathways with the highest correlation coefficients computed when evaluated on the test samples of the ‘breast cancer dataset’. It demonstrates that for these pathways, the correlation score between true and STGAT-predicted gene expression profiles is higher than the others. Through literature review, it was found that indeed, most of the top pathways have direct or indirect impacts on cancer or tumor cells [68–70].

To further investigate the performance of STGAT, we explored the effect of decreasing the number of genes involved in the analysis. Varying percentages (0.1%, 1.0%, 10% and 50.0%) of the total number of genes were randomly selected for the ‘breast cancer dataset’ and compared the correlation results in Supplementary Fig. S4. The experiment with each percentage value was repeated 10 times to draw the boxplots. This evaluation reveals valuable insights about the model’s behavior. Although a particular gene might not have any direct impact on another gene’s prediction, it contributes to the neighbor’s attention coefficient. Hence, when the number of genes is very low (0.1%, 1%), the performance of the model drops significantly along with an increase in the standard deviation. When 10% of the genes (approximately 1000 genes) are used, the model achieves optimum performance, signifying that with proper feature selection, the model can perform much better at this level. Further increases in the number of genes do not improve the correlation score; however, they decrease the standard deviation since the model becomes generalized over more genes.

#### Estimated gene expression distinguished the tumor and non-tumor spots

**Figure 4:**
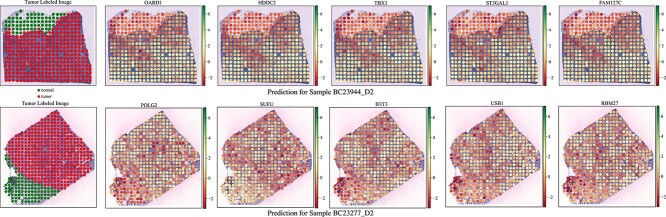
Examples of genes that distinguish the tumor and non-tumor tissue spots. The first image in each row illustrates the accurate label information for the tumor and non-tumor spots. The subsequent five heatmap images display the gene expression estimated by STGAT for the genes with the most significant $P$-values, comparing expressions between tumor and non-tumor spots.

As part of the analysis focusing on the gene expression generated by the SEG module, our objective is to assess whether the predicted gene expression contains enough information to differentiate between tumor and non-tumor tissue spots. For this experiment, we analyzed the predicted gene expression at the individual spot-level using STGAT for samples in the ‘breast cancer dataset.’ In the context of a single sample (image), top $100$ genes with the most variance are ranked based on their $t$-test $P$-value between tumor and non-tumor tissue spots. We created heatmaps displaying the five genes with the most significant $P$-values for two samples (appearing in two rows), as shown in Fig. 4. The first image in each row provides the true labeling of tumor and non-tumor tissue spots. These genes established connections to cancer as evidenced by previous research, are outlined in Table 1.

**Table 1:** Literature review of the candidate cancer genes. This table presents citations highlighting the relevance of the signature genes depicted in Fig. 4 to cancer for two patient samples in the ‘breast cancer dataset’

**Sample name**	**Gene name**	**Description**	**Reference**	** $P$ -value**
BC23944_D2	OARD1	Collectively prognostic, not studied individually yet	[71]	$5.9976e$ -$31$
	TBX1	High expression in tumor tissues; could promote cell proliferation,	[72]	$1.2222e$ -$30$
		migration, invasion, and cell cycle progression		
	FAM127C	Undergoes X-chromosome inactivation affecting cancers	[73]	$1.4752e$ -$30$
	ST3GAL1	Silencing suppresses tumor growth along with a notable decrease	[74]	$1.5358e$ -$30$
		in vascularity of MCF7 xenograft tumors		
BC23277_D2	POLG2	Somatic mutations frequently found in breast cancer;	[75]	$1.2008e$ -$18$
		decreases OXPHOS resulting in mtDNA depletion in breast tumors		
	SUFU	Suppresses RSL3-induced ferroptosis sensitivity of breast cancer cells;	[76]	$9.8341e$ -$15$
		regulates the Hippo pathway in breast cancer cells by interacting with LATS1		
	IFIT3	Classified as IRDS subset gene with better therapeutic response to IFNs;	[77]	$6.8357e$ -$13$
		hence considered predictive biomarker in many primary human cancers		
	RBM27	Capable of predicting overall survival in case of breast cancer	[78]	$2.6176e$ -$12$
	USB1	Affects growth of thyroid tumor cell lines; could induce cell cycle arrest in the	[79]	$2.6440e$ -$12$
		G1 phase, thereby suppressing cell proliferation and migration		

The results demonstrate that the gene expression predicted by STGAT can distinctly differentiate between tumor and non-tumor tissue spots. The differentially expressed genes are also associated with cancer according to previously established studies. For instance, the gene $TBX1$, known for its high expression in breast cancer tumor tissues [72], can effectively distinguish tumor and non-tumor spots. In addition, the prediction of $TBX1$ expression across the tumor image show a high-resolution expression pattern of $TBX1$: in non-tumor tissue area, its expression is low, and it gradually increases the expression pattern as the spot moves away from the non-tumor tissue area (Fig. 4 upper panel). Similarly, other genes predicted to increase their expression in sample- BC23944_D2 show a similar pattern. This observation suggests the potential of using this gene as a viable marker for a precise areal definition of tumor and therapeutic target for drug development. Similarly, the gene $POLG2$ is found to be mutated in $63$% of breast tumors [75]. However, its differential gene expression was not associated with breast cancer. Besides, some genes such as $IFIT3$ and $USB1$ that have not yet been linked to breast cancer could be promising candidates for future research directions, aiming to determine if they offer any prognostic insights into breast cancer.

#### Estimated gene expression improved cell-type classification

We also assessed the predictive performance of estimated gene expression using STGAT across various cell types within the same sample. This investigation involved eight samples from the ‘HER2+ dataset,’ as detailed cell-type information was available for these samples [54]. Each sample exhibited a distinct number of cell types, and a separate Support Vector Machine (SVM) model was trained and tested using true and predicted spot-level gene expression for each sample. The resulting AUROC scores are presented in Supplementary Table S4. Notably, in five out of the eight samples, the predicted gene expression demonstrated superior predictive power in distinguishing cell types compared to the true gene expression. This outcome underscores the efficacy of the spot-level gene expression generated by STGAT in facilitating downstream prediction tasks.

### Evaluation with TCGA Data

As demonstrated in the previous section, the SEG module exhibits remarkable predictive performance in gene expression estimation at the spot-level resolution (i.e. near cell level resolution) within spatial transcriptomic datasets. However, after its training on spatial transcriptomic data, the SEG module demonstrates limited efficacy when applied to WSIs and their corresponding bulk RNA-seq gene expression data, such as those sourced from TCGA studies. These data in large-scale disease studies introduce distinctive challenges when it comes to the prediction of gene expression at the spot-level: (i) absence of spatial coordinates: unlike spatial transcriptomic data, WSIs lack explicit spatial coordinate information, making it difficult to infer the precise spatial origin of gene expression. (ii) Lack of ground truth gene expression values: unlike spatial transcriptomic datasets, the dataset does not provide true gene expression values at the spot-level, further complicating the estimation process. (iii) Divergent expression distributions: the distribution of bulk RNA-seq gene expression and the gene expression patterns observed in spatial transcriptomic data are dissimilar, exacerbating the challenges of accurate gene expression prediction at the spot-level.

In order to address the challenges, we employ the GEP module introduced in the Materials and methods section. It leverages gene expression estimates obtained from bulk RNA-seq experiments to predict the corresponding gene expression levels at the spot-level. Specifically, following normalization, the gene expression estimated from bulk RNA-seq data serves as a guiding reference for spot-level gene expression predictions. Furthermore, the predicted tumor or non-tumor labels generated by the SLP module enable the proposed framework, STGAT, to focus solely on the gene expression values estimated from tumor spots, providing more accurate molecular signatures for downstream data analysis.

The STGAT model is trained to generate gene expression profiles for a total of 10 397 genes, which are overlapped between the spatial transcriptomic and TCGA datasets. To demonstrate the effectiveness and proficiency of the predicted spot-level gene expression for TCGA samples, we conducted a series of comprehensive downstream experiments.

#### STGAT improved breast cancer sub-type prediction

To evaluate the quality of the estimated spot-level gene expression by STGAT, we designed three cancer sub-type prediction tasks using 349 patient samples from the TCGA breast cancer dataset. These tasks were undertaken with the following assumptions: (i) the estimated gene expression data by STGAT achieves a near-cell-level resolution, offering more precise molecular signatures for cancer sub-type prediction in comparison to bulk RNA-seq gene expression data. (ii) Gene expression estimates derived solely from tumor spots provide superior predictive power for cancer outcomes when contrasted with gene expression data originating from a mixture of both tumor and non-tumor tissue samples. In this experiment, the STGAT model was trained on a spatial transcriptomics ‘breast cancer dataset,’ characterized by similar cancer sub-types as those present in the TCGA breast cancer samples.

There are 280 Estrogen Receptor positive (ER+) and 69 ER negative (ER-) samples, 239 Progesterone Receptor positive (PR+) and 110 PR negative (PR-) samples, and 110 Triple-negative (TN) and 294 non-TN samples in the dataset. The three tasks were to predict breast cancer patients’ ER, PR and TN statuses. The average gene expression of tumor spots, estimated by STGAT, and the average gene expression of all spots (tumor + non-tumor tissue), estimated by STGAT, are compared with the gene expression generated from bulk RNA-seq data. SVM was applied for the prediction task. The estimated gene expression data is divided into training and test sets, containing 80 and 20% of the total samples, respectively. We ran the classifiers on the three estimated gene expression datasets with the above-mentioned splitting 100 times. The average AUROC scores and their standard deviation (SD) for the 100 splits of the three datasets are reported in Table 2.

**Table 2:** Average AUROC scores and their SD for classifying TCGA breast cancer patients based on clinical variables using bulk RNA-seq gene expression data, the average gene expression of all spots (tumor + non-tumor tissue), and the average gene expression of tumor spots (tumor-specific average gene expression). The most significant AUROC scores are bolded. ‘*’ indicates that the difference between the results on the tumor-only spot and the other two cases is statistically significant ($P$-value < 0.0001)

**Sub-type**	**Gene Expression**	**AUROC**	**SD**
ER	bulk RNA-seq	$0.8776^{*}$	$0.0439$
	all spots	$0.8973^{*}$	$0.0494$
	tumor spots only	$\mathbf{0.9302}$	$0.0352$
PR	bulk RNA-seq	$0.8064^{*}$	$0.0519$
	all spots	$0.8125^{*}$	$0.0539$
	tumor spots only	$\mathbf{0.8514}$	$0.0506$
TN	bulk RNA-seq	$0.8962^{*}$	$0.0450$
	all spots	$0.9179$	$0.0475$
	tumor spots only	$\mathbf{0.9211}$	0.0419

The results clearly show that using tumor-specific average gene expression performs better than the other two datasets in all three comparisons. This demonstrates that STGAT has the capability to estimate gene expression at the spot-level and re-visit TCGA data to perform a more accurate downstream analysis for cancer sub-type prediction. The enhanced performance of the proposed framework can be largely attributed to the information aggregation mechanism within the SEG module. Specifically, it is well-established that spatially proximate spots or cells exhibit correlated characteristics, a phenomenon supported by the spatial relationships inherent in biological tissues. The GAT layer leverages this spatial information to assign greater attention to neighboring nodes in comparison to those located at a distance. Consequently, this selective attention mechanism facilitates the transfer of properties from proximal nodes to the target node, thus enhancing the network’s ability to capture spatially coherent gene expression patterns.

We also note that the average gene expression from all spots exhibits similar predictive power compared to gene expression from bulk RNA-seq experiments. This is because STGAT uses bulk RNA-seq gene expression for training the GEP module and also as a reference when creating the gene expression profiles for individual spots. The guidance from bulk RNA-seq data governs the predicted spot-level gene expression in achieving similar distribution, which enhances its prognostic capability.

To explore the impact of individual components within the STGAT framework on generating high-quality gene expression data for subsequent analysis, an ablation study was performed, and the findings are illustrated in Supplementary Fig. S5. The evaluation is conducted on gene expression data derived from various configurations of the STGAT framework for patients’ TN status prediction. Specifically, the study compares STGAT without the GAT layer in the SEG module (STGAT - GAT), STGAT without bulk RNA-seq gene expression guidance in the GEP module (STGAT - bulk), and STGAT without the z-score normalization step in the GEP module (STGAT - norm), in addition to the complete STGAT framework. The findings illuminate that the GAT layer exerts the most significant influence, followed by the bulk gene expression and normalization steps, respectively. This underscores the critical role of the GAT layer in generating high-quality spot-level gene expression for cancer sub-type prediction.

#### STGAT improved tumor stage prediction

Motivated by the improved cancer sub-type prediction achieved with expression data estimated by STGAT, our study extended to assess its effectiveness in predicting tumor stage. Accurate tumor stage prediction plays a pivotal role in customizing treatment strategies to meet each patient’s unique needs. Among the 349 TCGA breast cancer patients, 93 were classified as Stage I, 199 as Stage II, 43 as Stage III and 12 as Stage IV, with two patients lacking tumor stage information. We applied the same configuration for the training and test sets as in the cancer sub-type prediction task, employing two classification models, Random Forest and SVM, for this multi-class classification endeavor. Furthermore, we utilized the same three types of gene expression data, including true bulk RNA-seq gene expression, average gene expression from all the spots, and tumor-specific average gene expression, in our analysis and comparison. The results are presented in Table 3. It is evident that tumor-specific gene expression data outperforms the other two gene expression datasets in terms of its predictive capacity for tumor stage. The significant $P$-value obtained when comparing the classification results further demonstrates the improvement. This experiment highlights STGAT’s capability to effectively integrate the morphological features of images with the network information of spot locations to proficiently acquire the distinctive characteristics associated with various tumor stages.

**Table 3:** Average AUROC scores and their SD for classifying TCGA breast cancer patients into different tumor stages using bulk RNA-seq gene expression data, the average gene expression of all spots (tumor + non-tumor tissue), and the average gene expression of tumor spots (tumor-specific average gene expression). The most significant AUROC scores are bolded. ‘*’ indicates that the difference between the results on the tumor-only spot and the other two cases is statistically significant ($P$-value < 0.001)

**Classifier**	**Gene Expression**	**AUROC**	**SD**
Random Forest	bulk RNA-seq	$0.5686^{*}$	$0.0407$
	all spots	$0.6023^{*}$	$0.0336$
	tumor spots only	$\mathbf{0.6262}$	$0.0361$
SVM	bulk RNA-seq	$0.5971^{*}$	$0.0459$
	all spots	$0.5850^{*}$	$0.0490$
	tumor spots only	$\mathbf{0.6211}$	$0.0476$

#### STGAT improved stratification of observed survival time

**Figure 5:**
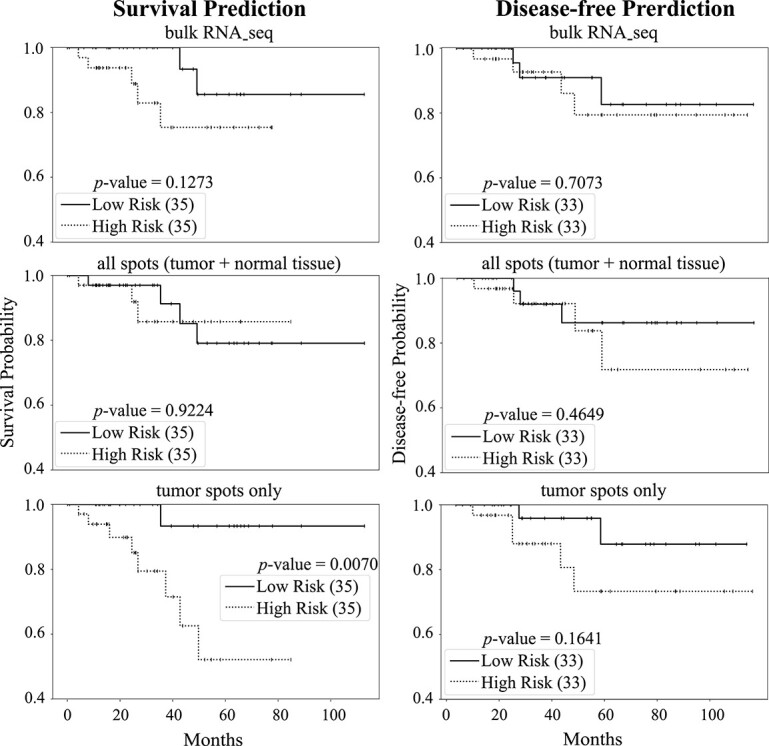
Survival and disease-free time predictions on TCGA breast cancer patients utilizing bulk RNA-seq gene expression and gene expression estimates derived from STGAT. KM plots illustrate low-risk (solid line) and high-risk (dashed line) groups based on the predicted prognostic index generated by bulk RNA-seq gene expression, the average gene expression of all spots (tumor + non-tumor tissue) estimated by STGAT, and the average gene expression of tumor spots estimated by STGAT for both survival and disease-free analyses. The number in parentheses indicates the sample count in the low- or high-risk group. The $P$-value is calculated using the log-rank test to compare the overall survival or disease-free probability of two groups of breast cancer patients.

To further assess the quality of spot-level gene expression data generated by STGAT, we conducted predictions on overall survival and disease-free time for TCGA breast cancer patients. The Cox proportional hazards model with elastic net penalty [80] was deployed to select gene expression predictive of patients’ outcome, i.e. overall survival or disease-free survival, including STGAT-estimated gene expression (tumor spots only and all spots) and bulk RNA-seq gene expression. 80% of the samples were utilized for training, and performance was tested on the remaining 20% of patient samples. The independent test set’s low and high-risk patient groups were generated based on the prognostic index [81]. Survival and disease-free predictions were visualized using Kaplan–Meier (KM) plots and compared using the log-rank test in Fig. 5. The Python packages *scikit-survival* [82] and *lifelines* [83] were employed for this analysis. The KM plots illustrate improved predictions of patient survival time and disease-free time based on the average expression of tumor spots compared to bulk RNA-seq gene expression or the average gene expression of all spots. Since the average gene expression of all spots and bulk RNA-seq gene expression include information from non-tumor spots, the message flow from these spots might impede performance. Log-rank test $P$-values further demonstrate the robust additional predictive power of the average gene expression of tumor spots beyond bulk RNA-seq gene expression.

**Figure 6:**
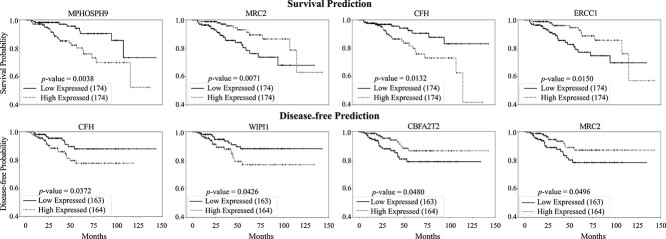
Survival and disease-free analysis based on gene expression level. The genes with the most significant log-rank test $P$-values computed between the low- and high-expressed patient groups are reported. This analysis utilized the average gene expression of tumor spots estimated by STGAT.

Furthermore, to identify the important genes that show the prediction power of patients survival and disease-free, we sorted patient samples for each gene according to their gene expression levels. Subsequently, these samples were divided into high and low expressed groups, and KM plots were created to visualize the survival and disease-free predictions for each group, as illustrated in Fig. 6. Only the top 100 genes with the greatest variances in gene expression are included in this analysis. The figure presents KM plots for four genes with the most significant log-rank test $P$-values. In this figure, the survival and disease-free curves distinctly show separation between patient groups with high and low gene expression for each gene, accompanied by the corresponding $P$-value. This result suggests that the gene expression predicted by STGAT provides clear and distinctive patterns, effectively distinguishing between breast cancer patient groups. Such outcomes are valuable for the selection of appropriate genes in diagnostic and analytical contexts, contributing to the development of targeted medical interventions.

## Discussion

Despite the availability of widespread genome sequencing data, detailed study of tissue sections at the single-cell level remains restricted due to the predominant use of bulk RNA-seq gene expression and H&E-stained WSIs in large-scale disease studies. While single-cell RNA-seq data provides cell-level gene expression, it lacks the spatial location information crucial for understanding tissue context in disease diagnosis and treatment. In contrast, spatial transcriptomics data combines gene expression at a near-cellular level with spatial location information. Despite the availability of bulk RNA-Seq data and WSIs in the TCGA consortium, the use of these highly profiled images and nucleotide-resolution level gene expression data is largely missing due to the lack of methodologies that could integrate this information together. In this study, we introduce STGAT, a machine learning framework trained on spatial transcriptomics data, enabling the prediction of expression for over 10,000 genes at the spot-level from TCGA WSIs. This allows STGAT to predict gene expression patterns with better resolution compared to bulk RNA-Seq. Additionally, STGAT maximizes its usability by applying the methodology to existing data.

During the gene expression prediction process, we encountered several challenges. Firstly, distribution disparities between TCGA bulk RNA-seq expression data and spatial transcriptomics gene expression data required the use of two weight matrices. These matrices, learned during training, were multiplied with spatial transcriptomics and TCGA gene expression data, ensuring alignment of the expression levels. Additionally, z-score normalization was applied before integrating the bulk TCGA gene expression vector into the spatial transcriptomics gene expression matrix. Secondly, an imbalance in the number of tumor and non-tumor spots in WSIs posed a challenge for training the SLP module to predict tumor/non-tumor labels. The Youden Index was employed to determine a threshold value for differentiating tumor and non-tumor spots. Thirdly, the variable number of spots in TCGA images (ranging from 150 to 4000) and the diverse networks created from different images required normalization of attention coefficients over all neighbors for a target spot, as detailed in *SEG* in the Materials and methods section.

STGAT demonstrated robust performance even with limited training data. The SEG module was trained on 42 spatial transcriptomics images, and the GEP module on 46 TCGA WSIs. Despite the limited data, STGAT’s generated gene expressions surpassed State-Of-The-Art baselines in evaluations for both spatial transcriptomics and TCGA data. On one hand, the predicted gene expression outperformed the baselines in terms of correlation with true gene expression (Figs 2 and 3). On the other hand, it rendered better results in the case of binary cancer sub-type prediction (Table 2), as well as multi-class tumor stage (Table 3) and cell-type prediction (Table S4) tasks. The capability of the predicted gene expression to effectively distinguish between tumor and non-tumor spots in Fig. 4 further validates the accuracy of the predicted gene expression profiles. Finally, the survival and disease-free prediction tasks, with significant $P$-values between high and low risk/expressed samples in Figs 5 and 6, demonstrate the reliability of the generated gene expression. Thus, STGAT can identify new cancer- and normal tissue-enriched transcripts based on a finer delineation of tumors and surrounding non-tumor tissues. This can discover previously unrecognized cancer-specific transcripts with spatial information that could be developed into new cancer markers for diagnosis and therapeutic assessments.

## Conclusion

Spatial transcriptomics has transformed the study of tissue structures, tumor composition and heterogeneity with detailed precision at the cellular level. However, due to its high cost, spatial transcriptomics technologies have not been widely used in large cohort studies. On the other hand, as genome sequencing methods have become more affordable, a large volume of omics data, along with corresponding whole slide images, is now accessible for large cohort studies. In this study, we introduced STGAT, a graph-based learning model that can estimate gene expression profiles at nearly the cellular level. We validate the predicted gene expression on TCGA breast cancer patient samples through various analyses, including cancer sub-type prediction, and survival and disease-free prediction, showcasing the effectiveness of STGAT. We believe that the gene expression profiles generated by STGAT at the spot-level show promise for developing targeted medicine and immunotherapy for various complex diseases.

**Key Points**

- A machine learning framework named STGAT has been developed to unravel the hidden information existing in bulk tissue data and predict gene expression profiles at near-cell (spot) level resolution.
- STGAT leverages Graph Attention Network to learn the relation and complex biological networks between the spots.
- After being trained on spatial transcriptomics data, the model outperformed state-of-the-art baselines in several experiments assessing the predictive capability.
- Further comprehensive experiments on TCGA bulk tissue data demonstrates that estimated gene expression provides more precise molecular signatures than true gene expression and hence can be utilized for better prognosis.

## Supplementary Material

STGAT_supp_bbae316

## Author Biographies

**Sudipto Baul** is currently a PhD student in Computer Science at the University of Central Florida. He completed his MS in Computer Science at the same university and received his BS in Electrical and Electronic Engineering from Bangladesh University of Engineering and Technology, Bangladesh. He is interested in developing computational methods to study the relationship and complex networks between biological entities and leverage the extracted information for better prognosis.

**Khandakar Tanvir Ahmed** is currently a PhD student in Computer Science at the University of Central Florida. He received his BS in Electronic Engineering from Bangladesh University of Engineering and Technology, Bangladesh. His research interests include modeling post-transcriptional regulations, and application of machine learning in computational biology.

**Qibing Jiang** is currently pursuing his PhD in Computer Science at the University of Central Florida. His research focuses on deep learning and computer vision, particularly on object detection and tracking. Before starting his PhD program, Qibing worked as a software engineer specializing in image and video processing. He holds a Master’s degree from Hanyang University in South Korea, where he researched image processing and video codec.

**Guangyu Wang** is an assistant professor at Houston Methodist, Weill Cornel Medical College. He received his PhD in Bioinformatics from Beijing Institute of Genomics, Chinese Academy of Sciences. His research is powered by artificial intelligence and machine learning to understand cell state transition and leverage pathology workflows by single-cell, spatial multi-omics and histopathology images.

**Qian Li** is an assistant member in St. Jude Children’s Research Hospital and Associate Member in St. Jude Comprehensive Cancer Center. Her research interest is statistical methods in multi-omics integration and longitudinal omics data in cancers and immunology.

**Jeongsik Yong** is an associate professor of Biochemistry, Molecular Biology and Biophysics at the University of Minnesota Twin Cities. He received his PhD and postdoctoral training in Biochemistry and Biophysics at the University of Pennsylvania. His research interests are broadly in the plasticity of functional transcriptomics by cellular signaling.

**Wei Zhang** is an associate professor and graduate program director in Computer Science at the University of Central Florida. He received his PhD in Computer Science from the University of Minnesota Twin Cities. His research interests are broadly in cancer genomics, network-based learning in bioinformatics; and disease phenotype prediction.

## References

[1] GTEx Consortium, Little AR, Moser MT. The Genotype-Tissue Expression (GTEx) pilot analysis: multitissue gene regulation in humans. Science 2015; 348:648–60. 10.1126/science.1262110. 25954001 PMC4547484

[2] Tomczak K, Czerwińska P, Wiznerowicz M. Review The Cancer Genome Atlas (TCGA): an immeasurable source of knowledge. Contemporary Oncology/Współczesna Onkologia 2015; 2015:68–77. 10.5114/wo.2014.47136 PMC4322527 25691825

[3] de Haan K, Zhang Y, Zuckerman JE. et al. Deep learning-based transformation of H&E stained tissues into special stains. Nat Commun 2021; 12. 10.1038/s41467-021-25221-2. PMC8361203 34385460

[4] Fridman WH, Pagès F, Sautès-Fridman C. et al. The immune contexture in human tumours: impact on clinical outcome. Nat Rev Cancer 2012; 12:298–306. 10.1038/nrc3245. 22419253

[5] Galon J, Costes A, Sanchez-Cabo F. et al. Type, density, and location of immune cells within human colorectal tumors predict clinical outcome. Science 2006; 313:1960–4. 10.1126/science.1129139. 17008531

[6] Li X, Wang C-Y. From bulk, single-cell to spatial RNA sequencing. Int J Oral Sci 2021; 13:36. 10.1038/s41368-021-00146-0. 34782601 PMC8593179

[7] Saliba A-E, Westermann AJ, Gorski SA. et al. Single-cell RNA-seq: advances and future challenges. Nucleic Acids Res 2014; 42:8845–60. 10.1093/nar/gku555. 25053837 PMC4132710

[8] Crosetto N, Bienko M, Van Oudenaarden. Spatially resolved transcriptomics and beyond. Nat Rev Genet 2015; 16:57–66. 10.1038/nrg3832. 25446315

[9] Marx V . Method of the year: spatially resolved transcriptomics. Nat Methods 2021; 18:9–14. 10.1038/s41592-020-01033-y. 33408395

[10] Rao A, Barkley D, França GS. et al. Exploring tissue architecture using spatial transcriptomics. Nature 2021; 596:211–20. 10.1038/s41586-021-03634-9. 34381231 PMC8475179

[11] Ståhl PL, Salmén F, Vickovic S. et al. Visualization and analysis of gene expression in tissue sections by spatial transcriptomics. Science 2016; 353:78–82. 10.1126/science.aaf2403. 27365449

[12] Berglund E, Maaskola J, Schultz N. et al. Spatial maps of prostate cancer transcriptomes reveal an unexplored landscape of heterogeneity. Nat Commun 2018; 9:2419. 10.1038/s41467-018-04724-5. 29925878 PMC6010471

[13] Thrane K, Eriksson H, Maaskola J. et al. Spatially resolved transcriptomics enables dissection of genetic heterogeneity in stage III cutaneous malignant melanoma. Cancer Res 2018; 78:5970–9. 10.1158/0008-5472.CAN-18-0747. 30154148

[14] Moncada R, Barkley D, Wagner F. et al. Integrating microarray-based spatial transcriptomics and single-cell RNA-seq reveals tissue architecture in pancreatic ductal adenocarcinomas. Nat Biotechnol 2020; 38:333–42. 10.1038/s41587-019-0392-8. 31932730

[15] Ji AL, Rubin AJ, Thrane K. et al. Multimodal analysis of composition and spatial architecture in human squamous cell carcinoma. Cell 2020; 182:497–514.e22. 10.1016/j.cell.2020.05.039. 32579974 PMC7391009

[16] Chen W-T, Ashley L, Craessaerts K. et al. Spatial transcriptomics and in situ sequencing to study Alzheimer’s disease. Cell 2020; 182:976–991.e19. 10.1016/j.cell.2020.06.038. 32702314

[17] Lundmark A, Gerasimcik N, Båge T. et al. Gene expression profiling of periodontitis-affected gingival tissue by spatial transcriptomics. Sci Rep 2018; 8:9370. 10.1038/s41598-018-27627-3. 29921943 PMC6008462

[18] Asp M, Salmén F, Ståhl PL. et al. Spatial detection of fetal marker genes expressed at low level in adult human heart tissue. Sci Rep 2017; 7:1–10. 10.1038/s41598-017-13462-5. 29021611 PMC5636908

[19] Maniatis S, Äijö T, Vickovic S. et al. Spatiotemporal dynamics of molecular pathology in amyotrophic lateral sclerosis. Science 2019; 364:89–93. 10.1126/science.aav9776. 30948552

[20] Maynard KR, Collado-Torres L, Weber LM. et al. Transcriptome-scale spatial gene expression in the human dorsolateral prefrontal cortex. Nat Neurosci 2021; 24:425–36. 10.1038/s41593-020-00787-0. 33558695 PMC8095368

[21] Saul D, Kosinsky RL. Spatial transcriptomics herald a new era of transcriptome research. Clin Transl Med 2023; 13. 10.1002/ctm2.1264. PMC10186148 37190941

[22] Weinstein JN, Collisson EA, Mills GB. et al. The Cancer Genome Atlas pan-cancer analysis project. Nat Genet 2013; 45:1113–20. 10.1038/ng.2764. 24071849 PMC3919969

[23] Sun S, Zhu J, Zhou X. Statistical analysis of spatial expression patterns for spatially resolved transcriptomic studies. Nat Methods 2020; 17:193–200. 10.1038/s41592-019-0701-7. 31988518 PMC7233129

[24] Svensson V, Teichmann SA, Stegle O. SpatialDE: identification of spatially variable genes. Nat Methods 2018; 15:343–6. 10.1038/nmeth.4636. 29553579 PMC6350895

[25] Elosua-Bayes M, Nieto P, Mereu E. et al. SPOTlight: seeded NMF regression to deconvolute spatial transcriptomics spots with single-cell transcriptomes. Nucleic Acids Res 2021; 49:e50–0. 10.1093/nar/gkab043. 33544846 PMC8136778

[26] Song Q, Jing S. DSTG: deconvoluting spatial transcriptomics data through graph-based artificial intelligence. Brief Bioinform 2021; 22:bbaa414. 33480403 10.1093/bib/bbaa414 PMC8425268

[27] Cang Z, Nie Q. Inferring spatial and signaling relationships between cells from single cell transcriptomic data. Nat Commun 2020; 11:2084. 10.1038/s41467-020-15968-5. 32350282 PMC7190659

[28] Biancalani T, Scalia G, Buffoni L. et al. Deep learning and alignment of spatially resolved single-cell transcriptomes with tangram. Nat Methods 2021; 18:1352–62. 10.1038/s41592-021-01264-7. 34711971 PMC8566243

[29] Jian H, Li X, Coleman K. et al. SpaGCN: integrating gene expression, spatial location and histology to identify spatial domains and spatially variable genes by graph convolutional network. Nat Methods 2021; 18:1342–51. 34711970 10.1038/s41592-021-01255-8

[30] Zhao E, Stone MR, Ren X. et al. Spatial transcriptomics at subspot resolution with BayesSpace. Nat Biotechnol 2021; 39:1375–84. 10.1038/s41587-021-00935-2. 34083791 PMC8763026

[31] Huazhu F, Hang X, Chong K. et al. Unsupervised spatially embedded deep representation of spatial transcriptomics. Genome Medicine 2024; 16(1):12. 10.1186/s13073-024-01283-x. PMC10790257 38217035

[32] He B, Bergenstråhle L, Stenbeck L. et al. Integrating spatial gene expression and breast tumour morphology via deep learning. Nat Biomed Eng 2020; 4:827–34. 10.1038/s41551-020-0578-x. 32572199

[33] Pang M, Kenong S, Li M. Leveraging information in spatial transcriptomics to predict super-resolution gene expression from histology images in tumors. bioRxiv 2021;2021–11. 10.1101/2021.11.28.470212.

[34] Zhang D, Schroeder A, Yan H. et al. Inferring super-resolution tissue architecture by integrating spatial transcriptomics with histology. Nat Biotechnol 2024:1–6. 10.1038/s41587-023-02019-9. 38168986 PMC11260191

[35] Gori M, Monfardini G, Scarselli F. A new model for learning in graph domains. In: Proceedings 2005 IEEE International Joint Conference on Neural Networks, volume 2, p. 729–34. IEEE, 2005.

[36] Scarselli F, Marco Gori A, Tsoi C. et al. The graph neural network model. IEEE Trans Neural Netw 2008; 20:61–80. 10.1109/TNN.2008.2005605. 19068426

[37] Zhang W, Chien J, Yong J. et al. Network-based machine learning and graph theory algorithms for precision oncology. NPJ Precis Oncol 2017; 1:25. 10.1038/s41698-017-0029-7. 29872707 PMC5871915

[38] Ahmed KT, Park S, Jiang Q. et al. Network-based drug sensitivity prediction. BMC Med Genomics 2020; 13:1–10. 10.1186/s12920-020-00829-3. 33371891 PMC7771088

[39] Wang H-Y, Zhao J-P, Yan-Sen S. et al. scCDG: a method based on DAE and GCN for scRNA-seq data analysis. IEEE/ACM Trans Comput Biol Bioinform 2021; 19:3685–94. 10.1109/TCBB.2021.3126641 34752401

[40] Dong K, Zhang S. Deciphering spatial domains from spatially resolved transcriptomics with an adaptive graph attention auto-encoder. Nat Commun 2022; 13:1739. 10.1038/s41467-022-29439-6. 35365632 PMC8976049

[41] Zeira R, Land M, Strzalkowski A. et al. Alignment and integration of spatial transcriptomics data. Nat Methods 2022; 19:567–75. 10.1038/s41592-022-01459-6. 35577957 PMC9334025

[42] Zeng Y, Wei Z, Weijiang Y. et al. Spatial transcriptomics prediction from histology jointly through transformer and graph neural networks. Brief Bioinform 2022; 23. 10.1093/bib/bbac297. 35849101

[43] Jia Y, Liu J, Chen L. et al. THItoGene: a deep learning method for predicting spatial transcriptomics from histological images. Brief Bioinform 2024; 25:bbad464. 10.1093/bib/bbad464 PMC10749789 38145948

[44] Veličković P, Cucurull G, Casanova A. et al. Graph attention networks. arXiv preprint arXiv:171010903 2017. 10.48550/arXiv.1710.10903.

[45] Baul S, Ahmed KT, Filipek J. et al. omicsGAT: graph attention network for cancer subtype analyses. Int J Mol Sci 2022; 23:10220. 36142140 10.3390/ijms231810220 PMC9499656

[46] Wen M, Zhang Z, Niu S. et al. Deep-learning-based drug–target interaction prediction. J Proteome Res 2017; 16:1401–9. 10.1021/acs.jproteome.6b00618. 28264154

[47] Long Y, Min W, Liu Y. et al. Ensembling graph attention networks for human microbe–drug association prediction. Bioinformatics 2020; 36:i779–86. 10.1093/bioinformatics/btaa891. 33381844

[48] Schapke J, Tavares A, Recamonde-Mendoza M. Epgat: gene essentiality prediction with graph attention networks. IEEE/ACM Trans Comput Biol Bioinform 2021; 19:1615–26. 10.1109/TCBB.2021.3054738 33497339

[49] Zhao J, Wang N, Wang H. et al. SCDRHA: a scRNA-seq data dimensionality reduction algorithm based on hierarchical autoencoder. Front Genet 2021; 12:733906. 10.3389/fgene.2021.733906. 34512734 PMC8429846

[50] Vaswani A, Shazeer N, Parmar N. et al. Attention is all you need. Adv Neural Inf Process Syst 2017; 30.

[51] Paszke A, Gross S, Massa F. et al. Pytorch: an imperative style, high-performance deep learning library. Adv Neural Inf Process Syst 2019; 32.

[52] Ruopp MD, Perkins NJ, Whitcomb BW. et al. Youden index and optimal cut-point estimated from observations affected by a lower limit of detection. Biom J 2008; 50:419–30. 10.1002/bimj.200710415. 18435502 PMC2515362

[53] Stenbeck L, Bergenstråhle L, Lundeberg J, Borg Å . Human breast cancer in situ capturing transcriptomics. Mendeley Data, V5, 2021. 10.17632/29ntw7sh4r.5.

[54] Andersson A, Larsson L, Stenbeck L. et al. Spatial deconvolution of HER2-positive breast cancer delineates tumor-associated cell type interactions. Nat Commun 2021; 12. 10.1038/s41467-021-26271-2. PMC8516894 34650042

[55] Chin L, Park PJ, Kucherlapati R. et al. Comprehensive molecular portraits of human breast tumours. Nature 2012; 490:61–70. 10.1038/nature11412. 23000897 PMC3465532

[56] Grossman RL, Heath AP, Ferretti V. et al. Toward a shared vision for cancer genomic data. New Engl J Med 2016; 375:1109–12. 10.1056/NEJMp1607591. 27653561 PMC6309165

[57] Goldman MJ, Craft B, Hastie M. et al. Visualizing and interpreting cancer genomics data via the Xena platform. Nat Biotechnol 2020; 38:675–8. 10.1038/s41587-020-0546-8. 32444850 PMC7386072

[58] Gao J, Aksoy BA, Dogrusoz U. et al. Integrative analysis of complex cancer genomics and clinical profiles using the cBioPortal. Sci Signal 2013; 6. 10.1126/scisignal.2004088. PMC4160307 23550210

[59] Chen C, Lu MY, Williamson DFK. et al. Fast and scalable search of whole-slide images via self-supervised deep learning. Nat Biomed Eng 2022; 6:1420–34. 10.1038/s41551-022-00929-8. 36217022 PMC9792371

[60] Dimitriou N, Arandjelović O, Caie PD. Deep learning for whole slide image analysis: an overview. Front Med 2019; 6:264. 10.3389/fmed.2019.00264. PMC6882930 31824952

[61] Aresta G, Araújo T, Kwok S. et al. BACH: grand challenge on breast cancer histology images. Med Image Anal 2019; 56:122–39. 31226662 10.1016/j.media.2019.05.010

[62] Janowczyk A, Madabhushi A. Deep learning for digital pathology image analysis: a comprehensive tutorial with selected use cases. J Pathol Inform 2016; 7:29. 10.4103/2153-3539.186902. 27563488 PMC4977982

[63] Dosovitskiy A, Beyer L, Kolesnikov A. et al. An image is worth 16x16 words: transformers for image recognition at scale. arXiv preprint arXiv:201011929 2020. 10.48550/arXiv.2010.11929.

[64] Hamilton W, Ying Z, Leskovec J. Inductive representation learning on large graphs. Adv Neural Inf Process Syst 2017; 30.

[65] Li C, Zhou A, Yao A. Omni-dimensional dynamic convolution. arXiv preprint arXiv:220907947 2022. 10.48550/arXiv.2209.07947.

[66] Mazzia V, Salvetti F, Chiaberge M. Efficient-capsnet: capsule network with self-attention routing. Sci Rep 2021; 11:14634. 10.1038/s41598-021-93977-0. 34282164 PMC8290018

[67] Kanehisa M, Goto S. KEGG: Kyoto Encyclopedia of Genes and Genomes. Nucleic Acids Res 2000; 28:27–30. 10.1093/nar/28.1.27. 10592173 PMC102409

[68] Grasmann G, Smolle E, Olschewski H. et al. Gluconeogenesis in cancer cells–repurposing of a starvation-induced metabolic pathway? Biochim Biophys Acta 2019; 1872:24–36. 10.1016/j.bbcan.2019.05.006. PMC6894939 31152822

[69] Zampieri M, Schoonvelde SAC, Vinci M, et al. Cancer treatment–related complications in patients with hypertrophic cardiomyopathy. In: Mayo Clinic Proceedings, volume 99, p. 218–28. Rochester, USA: Elsevier, 2024. 10.1016/j.mayocp.2023.10.003. 38180395

[70] Azaouagh A, Churzidse S, Konorza T. et al. Arrhythmogenic right ventricular cardiomyopathy/dysplasia: a review and update. Clin Res Cardiol 2011; 100:383–94. 10.1007/s00392-011-0295-2. 21360243

[71] Shimizu H, Nakayama KI. A 23 gene–based molecular prognostic score precisely predicts overall survival of breast cancer patients. EBioMedicine 2019; 46:150–9. 10.1016/j.ebiom.2019.07.046. 31358476 PMC6711850

[72] Huang S, Shu X, Ping J. et al. TBX1 functions as a putative oncogene of breast cancer through promoting cell cycle progression. Carcinogenesis 2022; 43:12–20. 10.1093/carcin/bgab111. 34919666 PMC8832409

[73] Larson NB, Fogarty ZC, Larson MC. et al. An integrative approach to assess X-chromosome inactivation using allele-specific expression with applications to epithelial ovarian cancer. Genet Epidemiol 2017; 41:898–914. 10.1002/gepi.22091. 29119601 PMC5726546

[74] Yeo HL, Fan T-C, Lin R-J. et al. Sialylation of vasorin by ST3Gal1 facilitates TGF-$\beta $1-mediated tumor angiogenesis and progression. Int J Cancer 2019; 144:1996–2007. 10.1002/ijc.31891. 30252131 PMC6590135

[75] Singh KK, Ayyasamy V, Owens KM. et al. Mutations in mitochondrial DNA polymerase-$\gamma $ promote breast tumorigenesis. J Hum Genet 2009; 54:516–24. 10.1038/jhg.2009.71. 19629138 PMC2782392

[76] Fang K, Sha D, Shen D. et al. SUFU suppresses ferroptosis sensitivity in breast cancer cells via hippo/YAP pathway. Iscience 2022; 25:104618. 10.1016/j.isci.2022.104618. 35800779 PMC9253713

[77] Pidugu VK, Pidugu HB, Meei-Maan W. et al. Emerging functions of human IFIT proteins in cancer. Front Mol Biosci 2019; 6:148. 10.3389/fmolb.2019.00148. 31921891 PMC6930875

[78] Groza I-M, Braicu C, Jurj A. et al. Cancer-associated stemness and epithelial-to-mesenchymal transition signatures related to breast invasive carcinoma prognostic. Cancer 2020; 12. 10.3390/cancers12103053. PMC7589570 33092068

[79] Ma Y, Yin S, Liu X-F. et al. Comprehensive analysis of the functions and prognostic value of RNA-binding proteins in thyroid cancer. Front Oncol 2021; 11:625007. 10.3389/fonc.2021.625007. 33816259 PMC8010172

[80] Yang Y, Zou H. A cocktail algorithm for solving the elastic net penalized Cox’s regression in high dimensions. Stat Interface 2013; 6:167–73. 10.4310/SII.2013.v6.n2.a1.

[81] Zhang W, Ota T, Shridhar V. et al. Network-based survival analysis reveals subnetwork signatures for predicting outcomes of ovarian cancer treatment. PLoS Comput Biol 2013; 9:e1002975. 10.1371/journal.pcbi.1002975. 23555212 PMC3605061

[82] Pölsterl S . Scikit-survival: a library for time-to-event analysis built on top of scikit-learn. J Mach Learn Res 2020; 21:1–6.34305477

[83] Davidson-Pilon C . Lifelines: survival analysis in python. J Open Source Soft 2019; 4:1317. 10.21105/joss.01317.

