# Supplementary material for: Integrating spatial transcriptomics and bulk RNA-seq: predicting gene expression with enhanced resolution through graph attention networks
Source: Brief Bioinform. 2024 Jul 3;25(4):bbae316. doi: 10.1093/bib/bbae316 (PMC11221891; doi:10.1093/bib/bbae316)
Supplement: STGAT_supp_bbae316 [file stgat_supp_bbae316.pdf]

Supplementary Document for "Integrating Spatial Transcriptomics and Bulk RNA-seq: Predicting Gene Expression with Enhanced Resolution through Graph Attention Networks"

Section S1: Evaluation Metrics

1. **Correlation score:** The pearson correlation coefficient between the true and predicted gene expression profiles was used as the evaluation metric for comparison between STGAT and the baseline methods following the formula:

$$\frac{\sum(y_i - \mu_i)(\widetilde{y}_i - \widetilde{\mu}_i)}{\sqrt{\sum(y_i - \mu_i)^2 \sum(\widetilde{y}_i - \widetilde{\mu}_i)}}$$

   where y_i is the true gene expression of a spot and $\widetilde{y}_i$ is the corresponding predicted gene expression. μ_i and $\widetilde{\mu}_i$ are their mean gene expressions, respectively. The correlation coefficient reveals the level of similarity and the direction of the relationship between two values. Therefore, it can be a good predictor of how similar the predicted gene expression values are to the true gene expression values.

2. **Mean Squared Error (MSE)**: This metric is also used to compare the performance of the models. It is computed between the true and predicted gene expression profiles following the formula:

$$||\widetilde{\mathbf{Y}}_i - \mathbf{Y}_i||_2^2$$

   where $\widetilde{\mathbf{Y}}_i$ and $\mathbf{Y}_i$ are the predicted and true gene expressions of a patient sample. MSE calculates the difference or distance between the two values. Therefore, MSE tells us how much difference is present between the predicted and true gene expression values. The correlation coefficient along with MSE together enables us to comprehend the closeness between the predicted and true data, thereby comparing the performance of the models.

3. **Area Under the Receiver Operating Characteristic curve (AUROC)**: This metric is used for comparison of the classification tasks on the TCGA data. It is defined as the area under the curve plotted using True Positive Rate (*precision*) along the y-axis and False Positive Rate (1-*specificity*) along the x-axis. It was implemented using *scikit-learn* [1] python package.

Section S2: Tables

Table S1: Notations for STGAT

Name	Definition
	SEG - Convolution Block
v	Number of spatial images
p	Number of genes to be predicted
$\mathbf{X}$	Set of all the spatial images
$\mathbf{X}_i$	Spatial image at the ith position
g_i	Number of spots in $\mathbf{X}_i$
e_c	Length of a convolution embedding vector for a single spot
$\mathbf{X}_{ij}$	Spot image at the jth position of the ith spatial image
$\mathbf{E}_{ij}^{(1)} \in \mathbb{R}^{e_c}$	Embedding vector generated from a CNN block for a single spot
$\mathbf{E}_i^{(1)} \in \mathbb{R}^{g_i \times e_c}$	Concatenated embedding of all the spots of image i
$\mathbf{E}_i^{(2)} \in \mathbb{R}^{g_i \times e_c}$	Embedding produced by linear block for image i
	SEG - GAT Block
e_a	Length of a GAT embedding vector
h	Number of heads in the GAT layer
f	A non-linear function
e	$= he_a$
$\mathbf{A}_i$	Adjacency matrix for image $\mathbf{X}_i$
$\mathcal{N}_{ij}$	Set of neighbors for the jth spot in the ith image
$\alpha_{n_{ijk}}$	Neighbor attention coefficient for the kth neighbor of jth spot of ith image
$\mathbf{a}_s, \mathbf{a}_n \in \mathbb{R}^{e_a}$	Self and neighbor attention vectors
$\mathbf{W}_s, \mathbf{W}_n \in \mathbb{R}^{e_a \times e_c}$	Self and neighbor weight matrices
$\mathbf{E}_{ij}^{(3)} \in \mathbb{R}^{e_a}$	j^{th} spot's GAT-embedding generated from a single head
$\mathbf{E}_{ij}^{(4)} \in \mathbb{R}^{e}$	Concatenation of embedding generated by all the heads for j^{th} spot
$\mathbf{Y}_i \in \mathbb{R}^{g_i \times p}$	Output prediction matrix for i^{th} image
	GEP Block
t	Number of WSIs
$\mathbf{T}$	Set of all the WSIs
$\mathbf{T}_i$	WSI at the ith position
m_i	Number of spots in $\mathbf{T}_i$
q	Length of bulk gene expression vector
$\widetilde{\mathbf{U}}$	Set of all the bulk gene expression vectors
$\widetilde{\mathbf{U}}_i \in \mathbb{R}^{q}$	Bulk gene expression vector for i^{th} WSI
$\mathbf{Z}_i^{(1)} \in \mathbb{R}^{e}$	i^{th} WSI's embedding after FC layer
$\mathbf{W}_{spots} \in \mathbb{R}^{e \times e}$	Learnable weight matrix for multiplication with $\mathbf{E}_i^{(4)}$
$\mathbf{W}_{bulk} \in \mathbb{R}^{e \times e}$	Learnable weight matrix for multiplication with $\mathbf{Z}_i^{(1)}$
$\mathbf{E}_i^{(5)}, \mathbf{Z}_i^{(2)}$	Products of corresponding multiplication
$\mathbf{Z}_i^{(3)} \in \mathbb{R}^{m_i \times e}$	Final embedding after addition of $\mathbf{E}_i^{(5)}$ and $\mathbf{Z}_i^{(2)}$
$\mathbf{Y}_i^{(2)} \in \mathbb{R}^{m_i \times p}$	Predicted gene expression matrix for i^{th} WSI

Table S2: Number of different blocks for each module. The table is organized in the computation sequence order.

Module	Block
SEG	6 × *Conv block*<br>1 × *Linear block*<br>1 × *GAT block*<br>3 × *Linear block*
GEP	2 × *Linear block*<br>2 × *Multiplier*<br>3 × *Linear block*
SLP	5 × *Conv block*<br>3 × *Linear block*

Table S3: Layers in each block. The number of heads in GAT block is a changeable hyperparameter.

Block	Layer
Conv block	Convolution + ReLU + MaxPool
Linear block	Linear + ReLU
GAT block	12 heads each with a graph attention layer

Table S4: Cell type classification. The spatial image spots from the eight samples in the 'HER2+ dataset' are individually categorized into distinct cell types (e.g., adipose tissue, invasive cancer, etc.) using SVM based on the predicted gene expression from STGAT.

Sample Name	Number of Classes	Gene Expression Type	AUROC	SD
A1	5	True	0.6932	0.0634
		Predicted	0.**7083**	0.1037
B1	4	True	0.6125	0.0430
		Predicted	0.**6796**	0.0347
C1	3	True	0.5170	0.0845
		Predicted	0.**7669**	0.0880
D1	3	True	0.**7232**	0.1123
		Predicted	0.5438	0.0898
E1	3	True	0.**8636**	0.0209
		Predicted	0.7884	0.0423
F1	3	True	0.**7753**	0.0479
		Predicted	0.7369	0.0320
G2	6	True	0.6142	0.0650
		Predicted	0.**6339**	0.0441
H1	6	True	0.6972	0.0251
		Predicted	0.**7909**	0.0304

Section S3: Figures

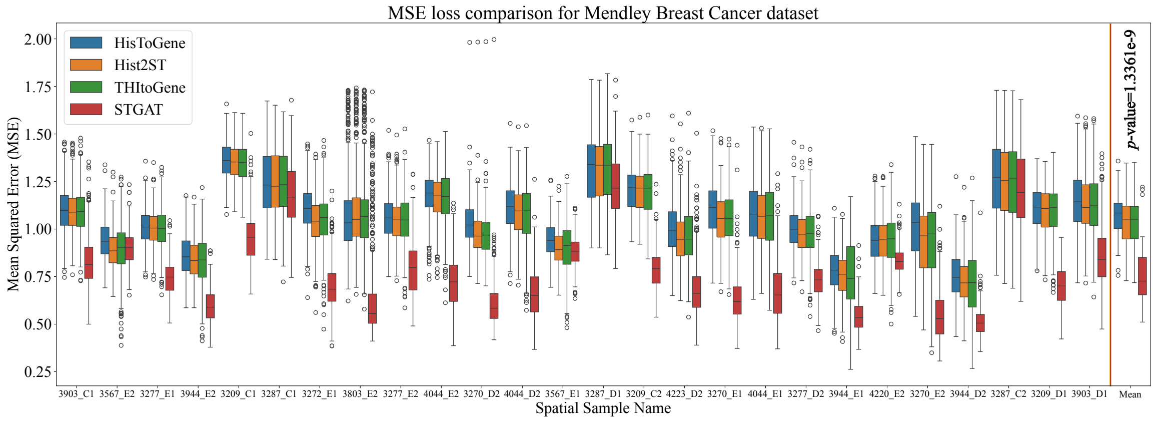

Figure S1: Comparison between STGAT and the baselines in terms of Mean Squared Error (MSE) loss computed between the predicted and true spot-level gene expression on the ‘breast cancer dataset’.

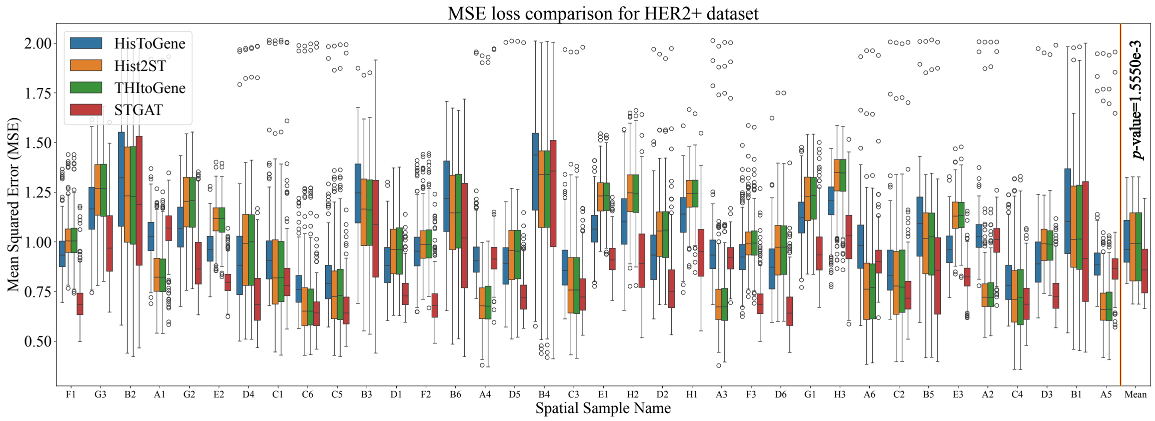

Figure S2: Comparison between STGAT and the baselines in terms of Mean Squared Error (MSE) loss computed between the predicted and true spot-level gene expression on the ‘HER2+ dataset’.

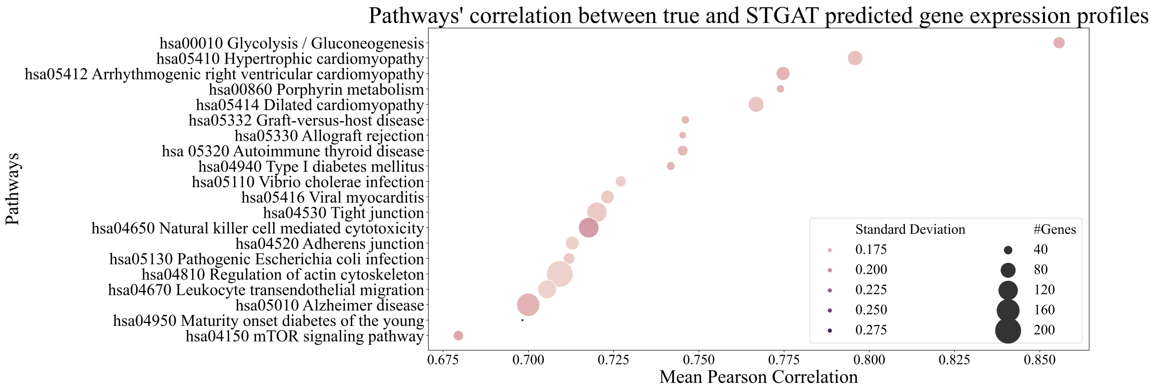

Figure S3: KEGG pathways with the highest mean Pearson correlation between the true and STGAT predicted gene expression profiles. The bubble size represents the number of genes contained in a pathway, and the color shades represent the standard deviation of correlation of the test samples.

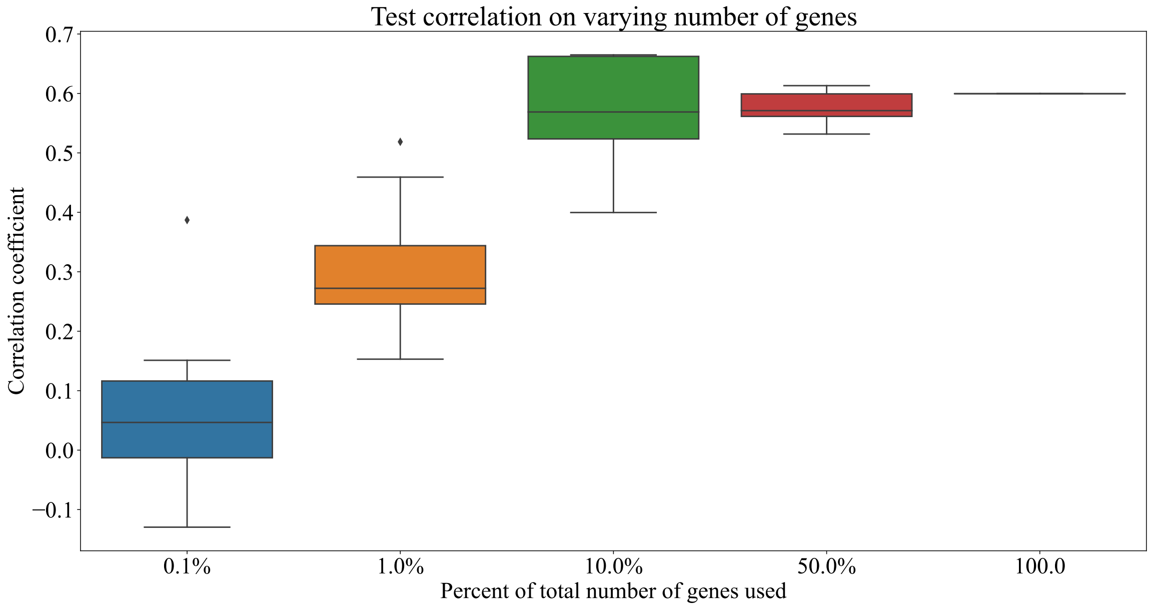

Figure S4: Correlation coefficient between true and generated gene expression on test samples with varying numbers of gene profiles used.

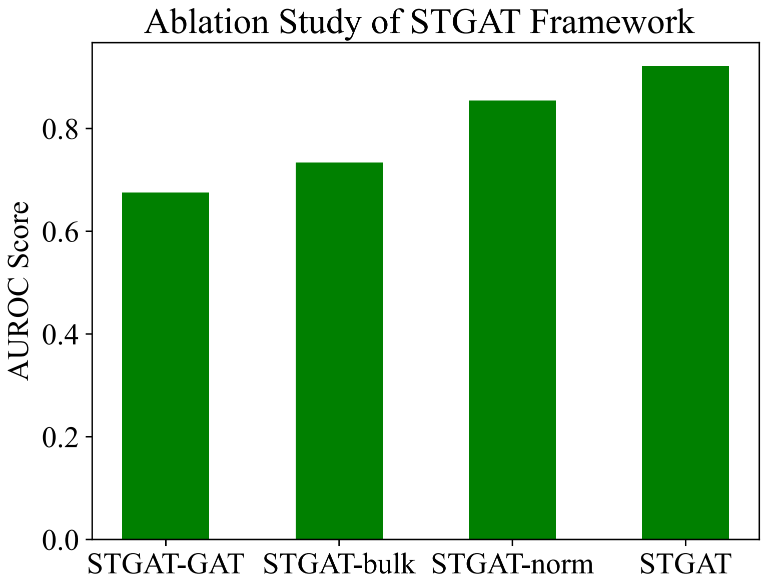

Figure S5: Ablation study of the STGAT framework. Performance comparison includes STGAT without the GAT layer in the SEG module (STGAT - GAT), STGAT without bulk RNA-seq gene expression guidance in the GEP module (STGAT - bulk), STGAT without the z-score normalization step in the GEP module (STGAT - norm), and the complete STGAT framework.

References

[1] Fabian Pedregosa, Gaël Varoquaux, Alexandre Gramfort, Vincent Michel, Bertrand Thirion, Olivier Grisel, Mathieu Blondel, Peter Prettenhofer, Ron Weiss, Vincent Dubourg, et al. Scikit-learn: Machine learning in Python. *the Journal of machine Learning research*, 12:2825–2830, 2011.
